# How slack resource affects hospital financial performance: The evidence from public hospitals in Beijing

**DOI:** 10.3389/fpubh.2022.982330

**Published:** 2022-09-15

**Authors:** Chen Chen, Xinrui Song, Junli Zhu

**Affiliations:** ^1^School of Public Health, Capital Medical University, Beijing, China; ^2^Research Center for Capital Health Management and Policy, Beijing, China; ^3^Beijing Chest Hospital, Capital Medical University, Beijing, China

**Keywords:** slack resources, financial performance, public hospital, Data Envelopment Analysis, return on assets, China

## Abstract

**Background:**

Beijing is a city with high concentration and congestion of quality medical resources in China. While moderate slack seems to be beneficial to the improvement of medical quality. The actual relationship between hospital slack resources and their performance deserves further exploration. The study aims to analyze the slack resources of public hospitals in Beijing and investigate the relationship between slack and hospital financial performance. Finding a reasonable range of slack to optimize resource allocation.

**Methods:**

The panel data of 22 public hospitals in Beijing from 2005 to 2011 were selected as the sample, and the DEA model was applied to measure the main variable using DEAP 2.1. Descriptive statistical analysis was performed using Excel and STATA 15. Pearson correlation coefficient analysis and variance inflation factor test were performed for each variable to avoid multicollinearity. The HAUSMAN test was used to determine the appropriate panel regression model, and then to analyze the influence relationship between the variables.

**Results:**

From 2005 to 2011, hospital slack resource transitioned from high to low. The slack measured by the DEA model has an inverted U-shaped relationship with financial performance, with ROA increasing from 4.088 to 8.083 when slack increases from 0 to about 0.378, and then showing a decreasing trend; slack measured by financial indicators has a transposed S-shaped relationship with financial performance, with ROA increasing when slack increase from 3.772 to 5.933.

**Conclusions:**

The slack resources of Beijing public hospitals decreased year by year from 2005 to 2011. Moderate slack resources are conducive to the improvement of healthcare quality, but when slack resources increase to a certain level, it will have a negative impact on healthcare quality. Therefore, hospital managers should control the slack within a moderate range according to the hospital operation policy and development plan to obtain the best performance.

## Introduction

According to figures from the World Health Organization, Global spending on health more than doubled in real terms over the past two decades, reaching US$ 8.5 trillion in 2019. Over the same period, global GDP increased by 74%, from US$ 50 trillion to US$ 86 trillion. Consequently, health spending as a share of global GDP rose from 8.5 to 9.8% (1). However, the increased investment in healthcare resources has not matched the public's expectations for healthcare services. As a result, discussions about the efficiency of resource use in health care organizations are taking place in a broader field. Campos et al. (2) analyzed the efficiency of the Spanish health system through the Data Envelopment Analysis (DEA) methodology and indicated that efficiency cannot be achieved without reducing health care expenses per resident and incrementing the number of primary medical care and nursing. Radojicic et al. (3) evaluated the efficiency of health systems in 38 countries, the main members of the Organization for Economic Co-operation and Development.

In China, public hospitals are the subject of the medical service system and bear the main responsibility for meeting the basic needs of the people for medical care. According to data from the China Health Statistics Yearbook, China's health spending per capita in 2019 was 4,702.8 Yuan (equivalent to US$696.96 today) (4). As the city with the highest concentration of quality medical resources in China, Beijing has a large number of famous doctors and hospitals, attracting patients from all over the country, resulting in the scope of medical services far beyond the administrative area. As the capital city, Beijing also leads the 31 regions in health spending per capita at 13,766.77 Yuan (equivalent to US$2164.14 today) (5), which is higher than the health spending per capita of 29 large high-income countries published by the World Health Organization (1). Yet public hospitals seem to have difficulty maintaining a balance between inputs and outputs (6). The abnormal health spending has reflected that people are facing costly medical care. Therefore, while increasing resource investment, we should also focus on budget, cost, procurement, and asset management. It is especially important to optimize financial performance management mechanism and improve financial performance. In the context of the new healthcare reform, public hospital managers should focus on financial performance management to lay the foundation for subsequent development (7).

Many researchers have confirmed that slack resources and healthcare quality are related (8, 9), which inspires us that it may be possible to improve the financial performance of public hospitals by utilizing slack resources. Slack resources was first defined by March and Simon (10) as organizational slack, that is, an excess of resources that a company can flexibly use to deal with changes of the environment. The organizational slack theory believes that slack resources are common in enterprises, non-profit organizations, and government departments. The differences in the organizations' possession of slack resources and their ability to deploy and utilize slack resources are the key to the differences in organizational performance and competitiveness. Influenced by the planned economic system, the public hospitals in China have long operated crudely, inefficiently allocating and utilizing the medical resources resulting in a certain degree of resource slack. If slack resources can be used to improve financial performance, it will not only facilitate the allocation of hospital resources, but also help hospitals improve their economic management. On the other hand, although public hospitals are not-for-profit healthcare organizations, their objectives are complex due to the multi-stakeholder and sensitivity to the political environment (11). Under the pressure of public and government regulation, hospitals are forced to find a balance between healthcare quality and efficiency, and slack plays a key role in this. So the relationship between slack and hospital financial performance needs to be explored in depth.

Since the 1960s, slack theory has been used in the study of corporate strategy. According to Cybert and March (12), slack resources can encourage people to try new strategies and projects that sometimes lead to good returns to the organization, which is uncommon in resource-constrained circumstances. Leibenstein (13) and Williamson (14) believe that slack is an unnecessary cost to the firm and an indicator of inefficiency. Several scholars have introduced organizational slack theory to the health field. Using the DEA, Miller and Adam (15) assessed organizational slack in US hospitals and Mogha et al. (16) assessed it in India public hospitals. Youn et al. (17) measured service quality by mortality rate and found that organizational slack was positively related to service quality in non-profit hospitals (17). Mohr andYoung found a curvilinear relationship between organizational slack and quality performance in primary care (8). Multiple linear regression were used by Ng and Wang (18) to demonstrate that slack in human resources could reduce patient dissatisfaction.

In previous studies, Scholars have given three views on the connotation of resources slack: one, principal-agent theory suggests that slack means wasteful and inefficient resources for the whole organization, and therefore detrimental to organizational performance (19–21). Second, organizational theory suggests that slack resource has a positive effect in that it serves as a buffer when the organization faces changes in the external environment, provides flexibility in the development of organizational strategies, and helps to improve organizational performance (22, 23). However, many studies also indicate that the relationship between slack resources and performance is complex, which is the third view: slack resources and performance are not simply linearly related. Agusti-Perez et al. (24) indicates that potential slack has a U-shaped and inverted U-shaped relationship with financial performance across time spans, respectively, and Bejarano et al. (25) suggested that the slack-performance link can be described as an inverted U-shaped curve independently of slack type. In China, Zhang et al. (26) constructed an inverted U-shaped between the slack and market performance, and only two research have been conducted to analyze organizational slack in the healthcare field. Huang and Liu (27) used DEA to measure organizational slack in hospitals using a sample of 10 hospitals in Shanghai, and Xiong et al. (28) proposed the need to maintain slack in healthcare resources from the perspective of healthcare demand. Few studies have demonstrated the relationship between slack and financial performance. So what role do slack resources actually play in public hospital finances? As a series of measures are adopted to pursue modernization of hospital financial management, does slack enhance or harm the financial performance? Given this, this paper intends to explore the impact of slack resource in hospitals on their financial performance under the framework of the organizational slack theory. Compared with previous studies, this study has the following novelties. First, the number of studies applying slack resources to hospitals is limited, especially in China, and we enrich the slack theory by implementing it in hospitals. Secondly, we consider the financial performance of hospitals, a topic rarely discussed in previous research on slack resources. Thirdly, the traditional methods usually use DEA to measure slack resources, but we choose both DEA and financial indicators, which is an innovation.

## Methods

### Data

In Beijing, the ownerships of public hospitals are various, including the central government, Beijing municipal government, district-level governments, state-owned enterprises, university and the military et al. There are big differences in the supervision of public hospitals with different owners. Considering that the number of tertiary public hospitals owned by the Beijing municipal government is the largest and its data is enough for empirical research, this research chooses Beijing municipal public hospitals as the research subject. This research used the annual financial statements of 22 municipal public hospitals from 2005 to 2011 as the data source, with a total of 154 observations, and the economic information contained in the annual financial statements was used to calculate the indicators.

### Variable

#### Dependent variable

This research considered financial performance as the dependent variable. As the prime purpose of business operation is profitability, many scholars take the data reflecting the profitability of enterprises as the most direct and powerful indicator to measure the performance of enterprises. Although public hospitals are non-profit organizations, due to the special nature of healthcare organizations, the hospitals still face the pressure of self-sufficiency, pressure from various stakeholders, and under the supervision of the public and government. So financial balance is also an important aspect of performance assessment that they have to consider carefully. Miller and Adam (15), Singh (29), and Bromiley (30) have commonly used “return on assets” (ROA) to measure the operational performance of firms. Vera et al. (31) also uses ROA as an indicator of hospital financial performance which is frequently used in the hospital sector. Therefore, the research selects ROA as the dependent variable to measure the performance.

#### Independent variables

To gain a more comprehensive and objective understanding of the slack resource situation in hospitals, this research measures slack resource using DEA models and financial indicators. DEA has been widely adopted by scholars to measure and analyze slack resources in public hospitals, such as Valdmains et al. (9), Miller and Adam (15) with a sample of US hospitals, Mogha et al. (16) with a sample of Indian public hospitals using DEA method to measure the slack resources within hospitals, Huang and Liu (27) used the DEA method to measure hospital slack resources of 10 hospitals in Shanghai, China. It is feasible to measure hospital slack resources using this method. There are various DEA models to estimate the efficiency score. CCR and BCC models are the most classical and commonly used. The CCR model is the first model of DEA, which was proposed by Charnes et al. in 1978 (32). The CCR model assumes that the returns to scale of production technology are constant. In reality, however, many units are not at the optimal production scale, so the technical efficiency derived by CCR model contains a component of scale efficiency. Then Banker et al. (33) extended the CCR model and proposed the BCC model. The BCC model assumes variable scale efficiency and can calculate the pure technical efficiency and scale efficiency values separately. It can reflect the technical efficiency level of the decision unit more accurately than the CCR model. Thus, the variable payoffs of scale and output-oriented BCC models were chosen for this study. The overall effective Decision-Making Units have the highest relative efficiency with an overall efficiency score of 1. The slack is set as 1-DEA efficiency score and named “Slack1” in the results. The input indicators are the number of practicing physicians, registered nurses, and actual beds, and the output indicators are the number of outpatient and emergency visits, hospital discharges and surgery visits. These indicators are also the most used indicators by scholars after Dong et al. (34) analyzed the indicators of 85 Chinese public general hospital efficiency DEA research papers. Scholars also widely use financial data to measure slack when exploring the impact of slack on financial performance. To make the slack measurement more reasonable, this research refers to Bourgeois (35) metrics based on financial data and uses three financial indicators: current ratio, gearing ratio and overhead to operating income ratio. Gomes and Ramaswamy (36) found that all 3 indicators are attributed to a single factor and have similar eigenvectors through principal component factor analysis. Therefore, the average of the 3 indicators was taken for measuring hospital slack, which was named “Slack2” in the results.

Duggan (37) used population density as a control variable when analyzing the behavior of non-profit hospitals. Cardinaels et al. (38) took number of beds as a control variable when exploring the development of hospital cost systems. When Unruh and Zhang (39) examined hospital nurse staffing and patient safety, they used number of beds and level of urbanization as control variables. Combining the experience of previous scholars and considering the influence of service quality, financial income and expenditure of municipal hospitals in Beijing, the research adds GDP per capita, the proportion of the elderly population, the level of urbanization, health expenditure as a percentage of general public budget expenditure, hospital size and resident population density were determined as control variables. Since the 22 municipal public hospitals included general and specialized hospitals, the control variables of average charge per bed day were introduced to control for their intrinsic variability.

### Research models

Referring to previous studies, the research constructs a panel regression model to analyze the impact of slack resource on performance in Beijing municipal hospitals, controlling for seven control variables: GDP per capita, the proportion of the elderly population, the level of urbanization, health expenditure as a percentage of general public budget expenditure, hospital size, average charge per bed day and resident population density, which shows as following:


(1) $${\text{LnPERF}}_{it}=\beta_{0}+\beta_{1}\times {\text{SLACK}}_{it}+\beta_{2}\times {\text{C}}_{it}+\varepsilon_{it}$$

(2) $$\begin{array}{l} {\text{LnPERF}}_{it}=\beta_{0}+\beta_{1}\times {\text{SLACK}}_{it}+\beta_{2}\times {\text{SLACK}}_{it}^{2}+\beta_{3}\\ \;\times C_{it}+\varepsilon_{it} \end{array}$$

(3) $$\begin{array}{l} {\text{LnPERF}}_{it}=\beta_{0}+\beta_{1}\times \;{\text{SLACK}}_{it}+\beta_{2}\times {\text{SLACK}}_{it}^{2}+\beta_{3}\\ \;\times {\text{SLACK}}_{it}^{3}+\beta_{4}\times C_{it}+\varepsilon_{it} \end{array}$$

where PERF represents the hospital's financial performance, SLACK represents the slack resource in hospital, C represents the control variable and it represents the data of the hospital i in year t.

A U-shaped or inverted U-shaped relationship exists if the change in *R*^2^ and β_2_ are significant when the quadratic model is compared to the linear model. Similarly, if the change in *R*^2^ and β_3_ are significant in the cubic model compared to the quadratic model and the linear model, then a transposed S-shaped relationship (three-stage relationship model) exists. This research used DEAP 2.1, Excel and STATA 15 software for the construction and analysis of panel models.

## Results

### Slack resource status

**Figure 1:**
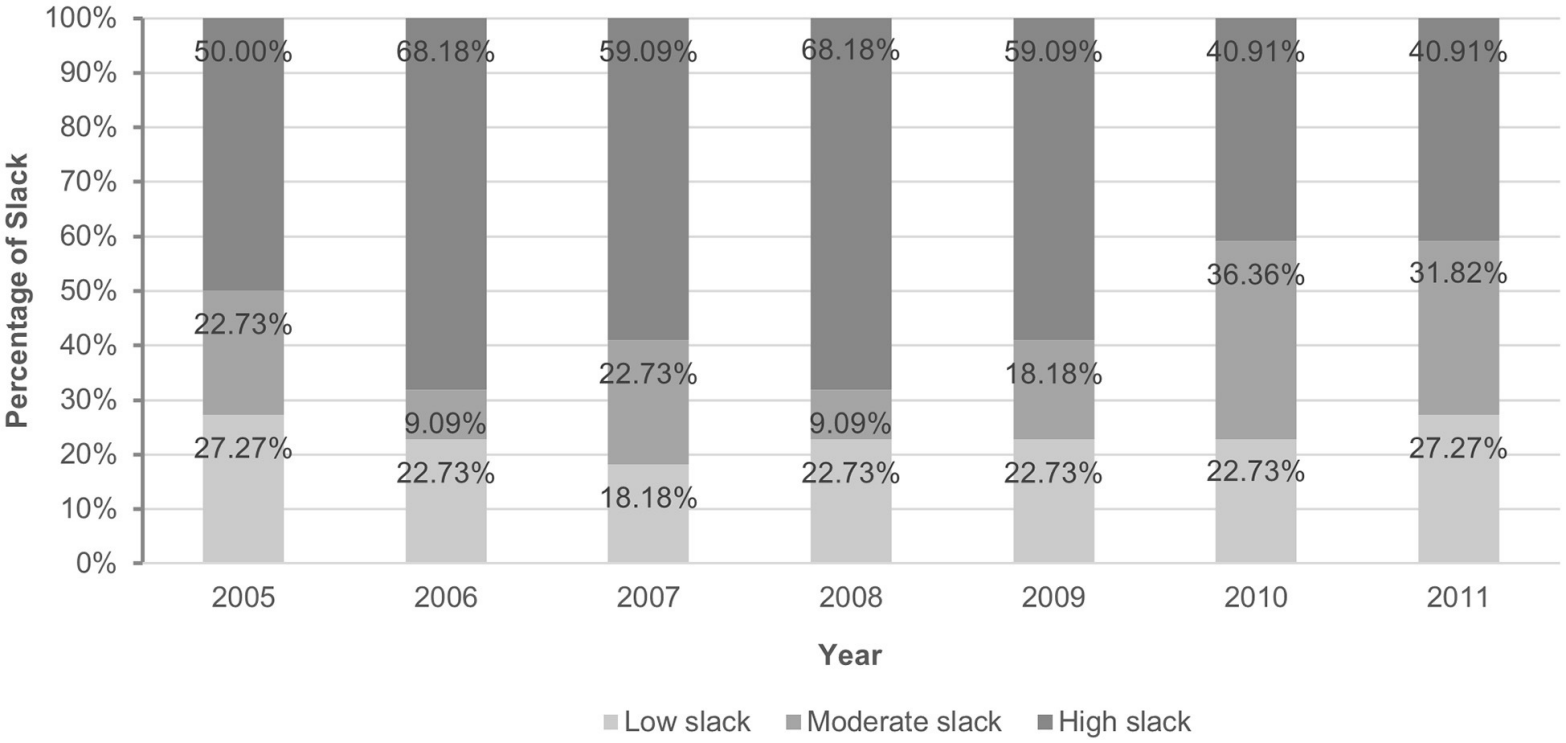
Status of slack resources in 22 hospitals.

Mehrtak et al. (40) classified DEA efficiency scores into high, moderate and low levels, based on which this research also classified slack resources into low, moderate and high slack, as shown in Figure 1. In 2006 and 2008, 22 public hospitals had the most severe slack resources, with 68.18% of hospitals in high slack. After this, the level of hospital slack gradually transferred to low. By 2011, the percentage of hospitals with high slack dropped to 40.91%, while the percentage of hospitals with low slack increased to 27.27%. In general, from 2005 to 2011, 22 hospitals showed a trend of transition from high to low slack. Guo et al. (55), when analyzing tertiary public hospitals in Beijing through DEA, also found that general hospitals had higher efficiency between 2009 and 2011, meaning that there were fewer slack resources.

### Correlation analysis and covariance test

To avoid heteroskedasticity, the GDP per capita, proportion of the elderly population, resident population density, the level of urbanization, health expenditure as a percentage of general public budget expenditure, the average charge per bed and hospital size were taken as logarithms. The Slack1 takes values in the range of (0, 1) and is not a ratio variable, so it is not taken as logarithm. ROA contains negative values and does not taken as logarithm.

According to Table 1, the mean, standard deviation, and correlation coefficients of the variables are presented. The Pearson correlation coefficient test indicates that the resident population density is significantly related to the GDP per capita (0.908) and the proportion of elderly population (0.882); the urbanization level is significantly related to the GDP per capita (0.814) and the resident population density (0.897), which may lead to multicollinearity problems in the regression models. Thus, variance inflation factor (VIF) was used to test for multicollinearity. The VIF value indicated that resident population density is multicollinear with other independent variables. After excluding this variable, there is no longer multicollinearity among the variables and regression analysis can be done.

**Table 1:** Correlation analysis of variables.

**Variables**	**Mean**	**SD**	**ROA**	**Slack1**	**Slack2**	**lngdppc**	**lnaging**	**lndensi**	**lnurban**	**lnexpen**	**lnmcr**	**lnbed**
ROA	5.728	8.082										
Slack1	0.433	0.285	−0.228^***^									
Slack2	4.605	0.589	0.397^***^									
lngdppc	11.26	0.625	0.178^**^	−0.319^***^	−0.191^***^							
lnaging	9.206	1.113	−0.013	−0.208^***^	−0.098	0.740^***^						
lndensi	2.025	0.194	0.087	−0.353^***^	−0.175^***^	0.908^***^	0.882^***^					
lnurban	6.484	0.669	0.042	−0.404^***^	−0.191^**^	0.814^***^	0.683^***^	0.897^***^				
lnexpen	5.728	8.082	0.018	−0.153^*^	0.053	−0.006	0.182^**^	0.175^**^	0.124			
lnmcr	4.605	0.589	0.064	−0.341^***^	−0.393^***^	0.238^***^	0.055	0.228^***^	0.279^***^	−0.046		
lnbed	2.418	0.242	−0.082	0.262^***^	−0.342^***^	−0.054	−0.149^*^	−0.115	−0.056	−0.113	0.213^***^	
lnsta	4.503	0.203	−0.075	−0.121	−0.300^***^	0.334^***^	0.252^***^	0.354^***^	0.375^***^	0.137^*^	0.469^***^	0.676^***^

N = 154; SD, standard deviation; ROA, return on assets; Slack1, slack measured by DEA; Slack2, slack measured by financial indicators; lngdppc, logarithm of the GDP per capita; lnaging, logarithm of proportion of the elderly population; lndensi, logarithm of resident population density; lnurban, logarithm of the level of urbanization; lnexpen, logarithm of health expenditure as a percentage of general public budget expenditure; lnmcr, logarithm of the average charge per bed; lnbed, logarithm of the number of beds; lnsta, logarithm of the total number of staff.

* p < 0.05.

** p < 0.01.

*** p < 0.001.

### Panel regression analysis

Panel regression models were used to analyze the linear, (inverted) U-shaped, and transposed S-shaped relationships between slack and ROA in municipal hospital. Model 1 to model 6 are constructed in turn. According to the results of the HAUSMAN test, a random-effects panel model was selected for models 4 and 5, and fixed-effects panel models were selected for the other models. Through the stray level regression of the robust standard, the results are shown in Table 2. Interestingly, slack1 and slack2 show different curves relationship with ROA. Slack1 and ROA: The quadratic coefficient of the slack resources in model 2 was significant at the 5% level (β = −27.870, *p* < 0.01), and its *R*^2^ was increased compared to model 1. The negative sign of β indicates that there is an inverted U-shaped relationship between slack and financial performance. Slack2 and ROA: The cubic coefficient of the slack resources in model 6 is significant at the 10% level (β = −2.845, *p* < 0.05) and the *R*^2^ increases compared to models 4 and 5, which indicating that the Slack2 and ROA are a transposed S-shaped relationship.

**Table 2:** Results from regression analyses.

**Variables**	**Model 1**	**Model 2**	**Model 3**	**Model 4**	**Model 5**	**Model 6**
Slack1	−2.140	21.061	−13.849			
(Slack1)^2^		−27.870^**^	81.959			
(Slack1)^3^			−81.745			
Slack2				7.789^***^	36.931^***^	−190.648
(Slack2)^2^					−3.012^***^	41.403^*^
(Slack2)^3^						−2.845^*^
lngdppc	7.353	7.219	6.495	9.030^***^	9.644^***^	10.666
lnaging	−18.976	−16.253	−13.662	−8.278^***^	−10.636^***^	−19.061^**^
lnurban	−1.575	−0.601	−2.791	−7.838	−8.180^*^	−12.688
lnexpen	3.363	4.806	5.082	6.794	6.524	2.471
lnbed	−19.589	−19.574	−19.301	3.658^***^	1.683^*^	−20.165
lnsta	−13.095	−13.851	−14.078	−4.887^***^	−3.215^**^	−8.302
lnmcr	7.349	7.955	10.543	3.632^***^	3.172^***^	5.940
Constant	140.082	126.308	120.361	−103.960^***^	−168.316^***^	410.788
*F*/Wald chi^2^	2.86^**^	4.27^***^	22.17^***^	53.69^***^	123.34^***^	5.14^***^
*R* ^2^	0.227	0.242	0.253	0.200	0.226	0.308

N = 154; Slack1, slack measured by DEA; Slack2, slack measured by financial indicators; lngdppc, logarithm of the GDP per capita; lnaging, logarithm of proportion of the elderly population; lndensi, logarithm of resident population density; lnurban, logarithm of the level of urbanization; lnexpen, logarithm of health expenditure as a percentage of general public budget expenditure; lnmcr, logarithm of the average charge per bed; lnbed, logarithm of the number of beds; lnsta, logarithm of the total number of staff.

* p < 0.05.

** p < 0.01.

*** p < 0.001.

**Figure 2:**
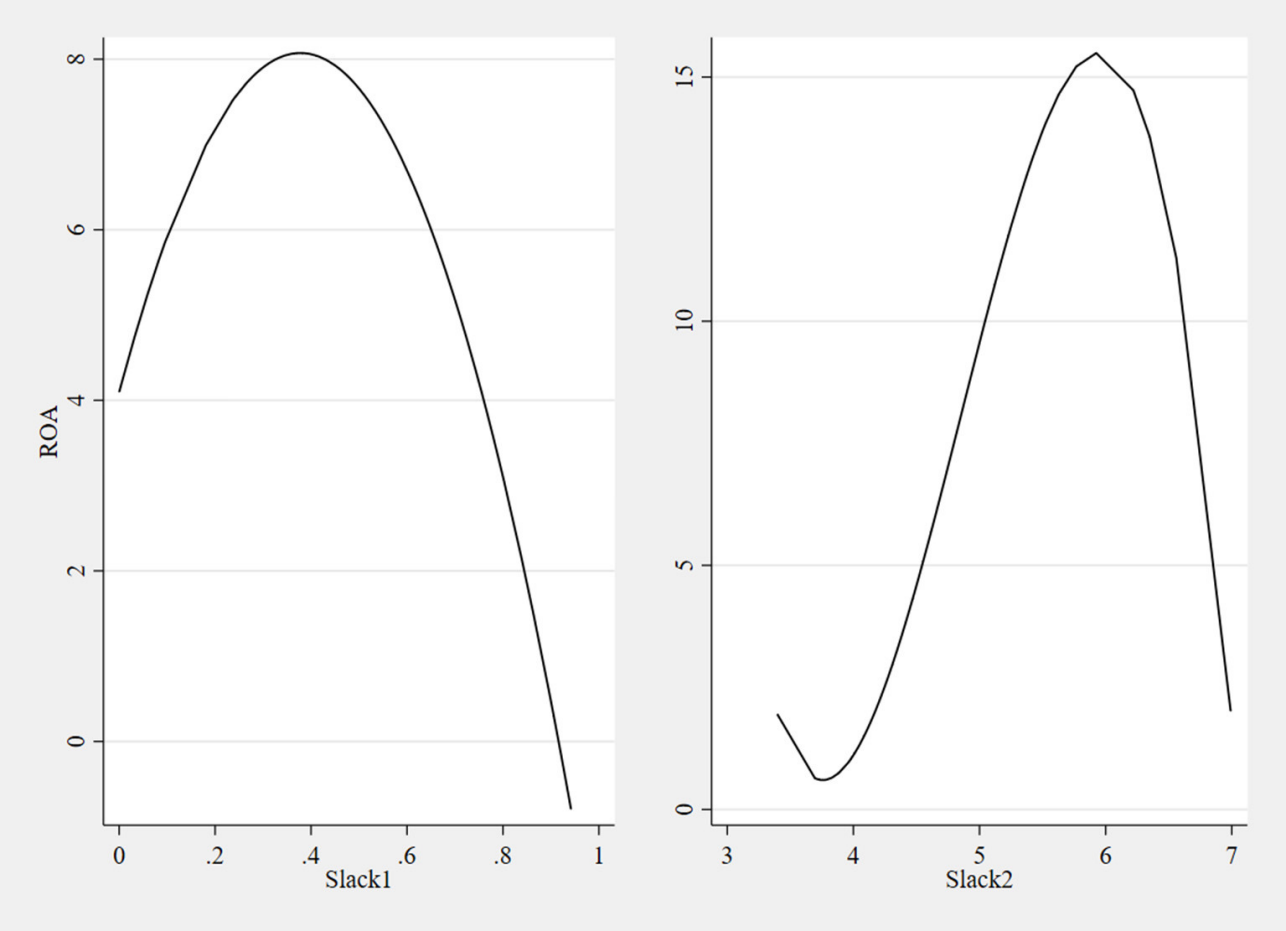
Regression fit plots for models 1–6.

The regression fits plots between Slack1, Slack2 and ROA were drawn by STATA 15 based on the panel regression model, as shown in Figure 2. The graph on the left shows that as Slack1 gradually increase from 0 to 0.378, the ROA increases from 4.088 to 8.083; as Slack1 increase to 0.943, the ROA decreases to −0.789. The right graph indicates that as the Slack2 increase from 3.402 to 3.772, the ROA decreases from 1.957 to 0.610; when the Slack2 increase to 5.933, the ROA increase to 15.496. When the Slack2 increase to 6.992, the ROA decreases to 2.001, which is close to the initial value of ROA when the Slack2 are 0.

Therefore, the results indicate that slack based on physical measures (Slack1) has an inverted U-shaped relationship with financial performance, and a low level of slack positively influences financial performance. Slack based on value measures (Slack2) has a transposed S-shaped relationship with financial performance, and a certain range of slack will contribute to financial performance.

## Discussion

In this research, the slack resources of 22 municipal public hospitals from 2005 to 2011 are measured. In 2005, the State Council published “Evaluation and Recommendations on the Reform of China's Medical and Health Care System”, which has evaluated that the equity of healthcare services in China had declined, the efficiency of healthcare investment was low, and China's healthcare reform was unsuccessful since the reform and opening-up. So the slack resources in this period were more obvious. With the initial conception, vigorous implementation, and gradual deepening of the New Medical Reform in 2009, the resource utilization efficiency of hospitals has been significantly improved, and high slack begin to transition to low slack. The research by Cui (41) also has indicated that the healthcare reform in Beijing from 2009 to 2011 has achieved significant results and that public hospitals are playing an important exemplary role as the main body of the health care system. The effectiveness of the healthcare reform is mainly reflected in the establishment of a new financial investment mechanism and the improvement of the public health funding mechanism, which makes the government's role in the medical field visible: in terms of the scope of input, the government is responsible for the infrastructure of public hospitals, the acquisition of equipment and the development of key disciplines; in terms of the way of input, the development and construction expenditures of public hospitals are funded by the government year by year. The above reforms have played a positive role in the strategic development of hospital and resource utilization, and the efficiency of hospitals has been significantly improved.

This research reveals an inverted U-shaped relationship between hospital financial performance and slack resource through DEA models, and the result is consistent with Nohria and Gulati (21) and Tan and Peng (42). Vitaliano (43)'s research shows that there is an economy of scale phenomenon in hospitals. Hospital efficiency increases with the expansion of scale, and when the optimal scale is reached, the marginal input-output efficiency begins to decline, showing a U-shaped cost function curve, which can well explain the inverted U-shaped relationship between hospital performance and slack resource in this paper. Holzhacker et al. (44) have suggested that congestion may not only have a serious adverse impact on important hospital outcomes such as mortality and morbidity but may also result in patients being turned away due to lack of capacity. So it's necessary to maintain slack resource. AgustÌ et al. (24) and Vanacker et al. (45) have reported that slack can act as an inducement to pay extra compensation for organizational members and as a buffer to help cope with environmental turbulence. Nguyen and Trinh (46) also have pointed out that slack can be effective in promoting organizational innovation and opening up new areas and markets, thus contributing to improved performance. But this does not mean that more slack is better: too much slack can make the organization satisfied with the status quo and thus become slow to perceive changes in the environment, and the cost of managing slack resources will increase. Although no such conclusion has been found in the studies of hospital organizations, it has been empirically confirmed in firms. For example, Kim et al. (47) have found too much slack in emerging economy firms may inhibit investment and decreased financial performance. After all, too much relaxation means inefficiency, which may lead to excessive growth of medical expenses. For public hospitals with public funding sources, it is not acceptable to regulators and the public. Therefore, the slack resources of public hospitals should be appropriate.

As mentioned above, besides measuring slack resources through the DEA model, this research also measures the slack through financial metrics and examines its impact on hospital performance. Interestingly, unlike the DEA model, it has a transposed S-shaped relationship with performance. Compared with the inverted U shape under the DEA model, it is negatively correlated with performance not only when there is more slack in public hospitals, but also less slack, and when slack resources grow to a certain level, its positive effects gradually become apparent. Sawyer et al. (48) and Stern et al. (49) have mentioned that organizations with little slack act more cautiously, and are reluctant to pursue good performance in a volatile but opportunity-filled environment. Greenley and Oktemgil (50) has also pointed out that taking strategic actions when there is less slack means that they may be at risk of declining performance. In other words, both less and more slack resources hurt financial performance, and only moderate slack is beneficial to financial performance (51). Although the results of the two methods of measuring slack are slightly different, there is considerable consistency in the results actually, and both demonstrate a non-linear relationship between slack and financial performance. Financial indicators are value measures, reflecting the economic status of the hospital, while DEA indicators are physical measures, reflecting the status of the hospital's healthcare resources.

## Conclusions

### Main findings

There is a significant impact of slack resources on financial performance, but the impact varies based on the level of slack. In light of government's continued investment in hospitals, rational use of slack resources is becoming increasingly important to improve hospitals' financial performance. The main findings of this research are summarized as follows.

(i) From 2005 to 2011, Beijing public hospitals had slack resources, which gradually transitioned from high to low levels after 2008. Other scholars have also come up with such results, suggesting that the efficiency of resource utilization in hospitals has improved after the 2009 healthcare reform.
(ii) Slack measured by the DEA model has an inverted U-shaped relationship with hospital performance, while slack measured by financial indicators has a transposed S-shaped relationship with financial performance. Both measures indicate that slack and financial performance are not simply linearly related.
(iii) Moderate slack is beneficial to the improvement of financial performance, but when slack resources increase to a certain degree, it will turn into a negative effect.

### Theoretical implications

This study applies slack theory to hospital performance management, especially financial performance, which has received little attention. The contribution of this study to the existing literature is as follows.

(i) Existing literature typically applies slack theory to business management, and even when applied to healthcare, it emphasizes service quality. This research applies slack theory to public hospitals and explores the impact of slack resources on financial performance, which is an enrichment and extension of slack theory.
(ii) In this paper, two methods are presented to measure slack in hospitals. One method is a value measure, which is better suited to for-profit organizations and should be applied with caution by not-for-profit hospitals. We can also obtain slack by using physical measures through the DEA model, which can take into account the characteristics of public hospitals. Both methods are objective and can be validated against each other, resulting in more reliable results.

### Practical implications

This study has practical implications for China as well as other countries around the world, which we summarize as follows.

(i) For China
  As the government continues to invest significant funds and resources into healthcare institutions, the corresponding measures for optimizing resource allocation should be refined to avoid creating excessive slack. The public hospitals should control slack within a moderate range according to the actual operation, government reform policies and future development plan to obtain the best performance.
(ii) For other countries
  Previous studies have mostly focused on public hospitals or non-profit hospitals in developed countries such as the US and the UK (52–54), which have large differences in hospital operations from China. Public hospitals are the major part of China's medical service system and are government-led organizations that embody public welfare, undertake basic medical care, and relieve people's difficulties in accessing medical care. The large population makes China's hospitals face greater pressure to provide health services. This paper provides experiences for countries with similar health systems that are facing the same challenges. As an emerging economy, China is undergoing an economic transition, and this paper also provides evidence for countries that are in the same period or will be emerging economies.

### Future work

This study can be extended through the following perspectives. First, this research examines hospitals' financial performance because public hospitals are under pressure to be self-sustaining, and financial income and expenditure are important performance aspects. Hospital performance is multidimensional, which can be included in subsequent studies for multidimensional analysis of healthcare quality, innovation, and social performance. Second, Nohria and Gulati (21) have shown that good performance also increases slack, indicating that the performance-slack relationship is dynamic. The dynamic relationship between slack and performance can be explored in depth in the future. Last, this research only chooses data from 2005 to 2011, one reason being that since 2005, health care reform began to be conceived, introduced, and implemented. Using data from this period for analysis can reveal the effectiveness of the reform. Another reason is the implementation of the new accounting system for hospitals in 2012, which has affected hospital financial accounting. This study uses data up to 2011 to ensure data comparability. Analysis of data after 2012 can be carried out later.

## Data availability statement

The raw data supporting the conclusions of this article will be made available by the authors, without undue reservation.

## Author contributions

JZ formulated the research concept and developed the primary framework of the study. Analysis for this study was conducted by XS, with assistance from CC. Interpretation of the results and preparation of the manuscript were completed by CC, XS, and JZ. Review and editing of the manuscript was completed by CC and JZ. CC and XS contributed equally to the final manuscript. All authors contributed to the manuscript revision, read, and approved the submitted version.

## Funding

This study was supported by the National Natural Science Foundation of China (Grant Nos. 71974133 and 71573182).

## Conflict of interest

The authors declare that the research was conducted in the absence of any commercial or financial relationships that could be construed as a potential conflict of interest.

## Publisher's note

All claims expressed in this article are solely those of the authors and do not necessarily represent those of their affiliated organizations, or those of the publisher, the editors and the reviewers. Any product that may be evaluated in this article, or claim that may be made by its manufacturer, is not guaranteed or endorsed by the publisher.
